# An economic evaluation of a specialist preventive care clinician in a community mental health service: a randomised controlled trial

**DOI:** 10.1186/s12913-020-05204-7

**Published:** 2020-05-11

**Authors:** Caitlin Fehily, Rod Ling, Andrew Searles, Kate Bartlem, John Wiggers, Rebecca Hodder, Andrew Wilson, Kim Colyvas, Jenny Bowman

**Affiliations:** 1 School of Psychology, Faculty of Science and Information Technology, The University of Newcastle, Callaghan, NSW Australia; 2 Hunter Medical Research Institute, Clinical Research Centre, New Lambton Heights, NSW Australia; 3 The Australian Preventive Partnership Centre (TAPPC), Sax Institute, Ultimo, NSW Australia; 4 School of Medicine and Public Health, Faculty of Health and Medicine, The University of Newcastle, Callaghan, NSW Australia; 5 Population Health, Hunter New England Local Health District, New Lambton, NSW Australia; 6 School of Mathematical and Physical Sciences, Faculty of Science and Information Technology, The University of Newcastle, Callaghan, Australia

**Keywords:** Chronic disease, Mental illness, Preventive care, Community mental health, Cost-effectiveness, Risk behaviours

## Abstract

**Background:**

Clinical practice guidelines and policies direct community mental health services to provide preventive care to address chronic disease risks, however, such care is infrequently provided in routine consultations. An alternative model of care is to appoint a clinician to the dedicated role of offering and providing preventive care in an additional consultation: the ‘specialist clinician’ model. Economic evaluations of models of care are needed to determine the cost of adhering to guidelines and policies, and to inform pragmatic service delivery decisions. This study is an economic evaluation of the specialist clinician model; designed to achieve policy concordant preventive care delivery.

**Methods:**

A retrospective analysis of the incremental costs, cost-effectiveness, and budget impact of a ‘specialist preventive care clinician’ (an occupational therapist) was conducted in a randomised controlled trial, where participants were randomised to receive usual care; or usual care plus the offer of an additional preventive care consultation with the specialist clinician. The study outcome was client acceptance of referrals to two free telephone-based chronic disease prevention services. This is a key care delivery outcome mandated by the local health district policy of the service. The base case analysis assumed the mental health service cost perspective. A budget impact analysis determined the annual budget required to implement the model of care for all clients of the community mental health service over 5 years.

**Results:**

There was a significantly greater increase from baseline to follow-up in the proportion of intervention participants accepting referrals to both telephone services, compared to usual care. The incremental cost-effectiveness ratio was $347 per additional acceptance of a referral (CI: $263–$494). The annual budget required to implement the model of care for all prospective clients was projected to be $711,446 over 5-years; resulting in 2616 accepted referrals.

**Conclusions:**

The evaluation provides key information regarding the costs for the mental health service to adhere to policy targets, indicating the model of care involved a low per client cost whilst increasing key preventive care delivery outcomes. Additional modelling is required to further explore its economic benefits.

**Trial registration:**

ACTRN12616001519448. Registered 3 November 2016, https://www.anzctr.org.au/Trial/Registration/TrialReview.aspx?id=371709.

## Background

People with a mental illness are more likely to engage in chronic disease risk behaviours, including: tobacco smoking, insufficient nutrition, harmful alcohol consumption, and physical inactivity, compared to the general population [1–5]. The higher prevalence of chronic disease risk behaviours contributes substantially to the greater burden of chronic disease morbidity and mortality [6–9], and reduced life expectancy [7, 10] experienced by people with a mental illness. The economic burden of chronic disease is considerable in Australia [2] and internationally [11], accounting for 36% of direct healthcare costs in Australia [2]. Given the considerable health and economic burden associated with chronic disease, there is a need to develop preventive interventions to redress the health and economic inequity experienced by this high-risk population group [12].

Clinical practice guidelines recognise that mental health services provide an opportunity to deliver evidence-based ‘preventive care’: care that aims to address clients’ chronic disease risk behaviours (tobacco smoking, poor nutrition, harmful alcohol consumption, and physical inactivity) [13–16]; which may comprise three care elements: assessment, brief advice, and referral [17–19]. Referral is an important preventive care outcome, with referral of clients to specialist chronic disease prevention services representing a key component of State and Health District-wide policies; and being a key performance indicator for health services, including mental health services [20–22]. Referral to specialist services is a fundamental component of health care delivery, and ensures clients receive ongoing, specialist care to support positive changes in risk behaviours. Referral to telephone-based chronic disease prevention services represents an important pillar of chronic disease prevention policy in New South Wales (NSW), Australia [20–22].

Provision of preventive care, including referral, has consistently been reported to be effective in reducing chronic disease risk behaviours [23–29]. However, low provision of evidence-based preventive care in routine mental health consultations has been reported in Australia [30–33] and internationally [34–37]. To address this, an alternative model of preventive care delivery has been suggested: implementing a dedicated clinical position to offer and provide clients with preventive care. While the exact role undertaken by a ‘specialist preventive care clinician’ has varied in previous research, and could be to encourage and supplement the provision of preventive care by usual clinicians in routine mental health consultations [38]; in three [39–41] of the four [38–41] previous studies identified, the role of the clinician was to offer preventive care to clients in an additional consultation. Of these three studies, two (a cross-sectional study [40] and a randomised controlled trial [39]) reported outcomes regarding preventive care provision, both demonstrating a positive intervention effect. In the cross-sectional study, the proportion of clients receiving metabolic monitoring was significantly higher in a service where this was the responsibility of a nurse in a dedicated role (78%), compared to a service where this care was provided by usual clinicians in routine consultations (3%; *p* = 0.01) [40]. The study did not consider the cost-efficiency or affordability of the model of care. Economic evaluations of models of service delivery that compare cost of delivery to a relevant outcome are both recommended and necessary [42, 43]. In an applied setting, these evaluations are required to determine the resources required to achieve the aims of guidelines and health policies. This information is then used to inform the pragmatic decisions of policy makers and service providers.

The second study, a randomised controlled trial in the US, examined the effectiveness [39], cost-effectiveness and budget impact [44] of the specialist clinician model of care. Clients were randomly allocated to receive usual care or additional consultations with a ‘care manager’ (a registered nurse) who had the role of providing education, advice, and assistance in overcoming barriers to accessing primary medical care. At a 2-year follow-up, the proportion of recommended preventive services clients received (measured across four domains: physical examinations, screening tests, vaccinations, and health education); was higher in the intervention group (56.2%) than control group (17.4%; *p* < 0.001). The average annual cost of the intervention per participant was US$973. The study also considered the budget impact of the intervention, an analysis which considers the expected financial, or accounting, impact to health services from implementing a new intervention in a health care system or service [45, 46]. When examined from this perspective, the intervention was not considered feasible to implement as revenues generated from Medicaid rebates did not cover the implementation costs. No previous research has explored the cost-effectiveness or budget impact of the model of care in other jurisdictions.

The applied study reported here was undertaken to inform decision makers about the resources needed to adhere to key policy imperatives. The aim of the study was to assess the costs, cost-effectiveness and budget impact of implementing the specialist preventive care clinician model of care to increase client acceptance of referrals to telephone-based chronic disease prevention services (the Get Healthy Service and Quitline). The evaluation was undertaken in a randomised controlled trial, where a ‘specialist preventive care clinician’ (an occupational therapist and experienced mental health clinician) was dedicated to the role of offering and providing clients of a community mental health service with an additional preventive care consultation [47].

## Methods

### Intervention trial design, setting, and sample

**Fig. 1:**
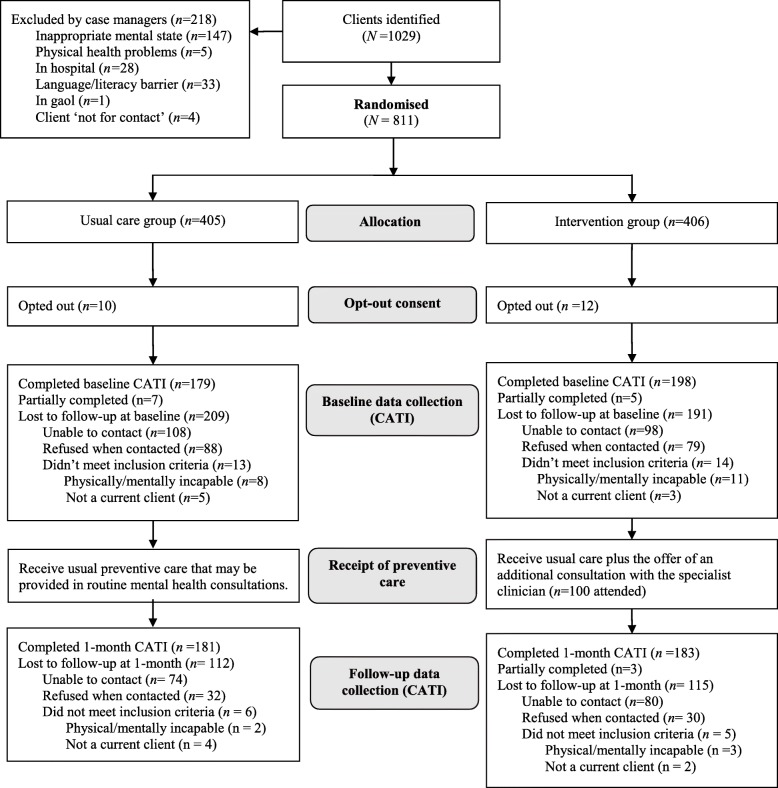
Trial design and participant flow through the trial

A two-group parallel randomised controlled trial was undertaken in one community mental health service in regional NSW (New South Wales) Australia (Fig. 1). The community mental health service is located in a NSW local health district that has a policy to provide preventive care (assessment, advice, and referral) for key risk behaviours in routine mental health consultations. A key component of the policy is referral to specialist chronic disease prevention services, with referrals directed to free, NSW telephone-based services: Get Healthy Service (for insufficient nutrition, harmful alcohol consumption, and physical inactivity) and Quitline (for tobacco smoking). These services have been reported to be effective in reducing risk behaviours [48, 49]. Provision of preventive care, including referral, under this policy has been sub-optimal [30, 31], hence the need for health service strategies to remedy this situation. A published protocol has described the methods for the study, which aimed to increase the provision of guideline and policy-concordant preventive care [47].

Clients eligible for the study (over the age of 18 and clinically determined by the treating team as sufficiently physically and mentally capable to participate) were identified by mental health service staff. All eligible clients were randomly allocated (1:1 ratio; block sizes 2, 4, and 6; generated by a statistician independent of the project) to usual care (preventive care provided by mental health clinicians in routine consultations) or usual care plus the offer of an additional consultation with a specialist preventive care clinician (intervention condition). For intervention participants, the additional consultation was scheduled to occur between the baseline and 1-month follow-up. Neither clients nor clinicians were blinded to study allocation, to align with how this model would be implemented. Outcome data were obtained via telephone interviews with participants at baseline (February–September 2017) and a 1-month (March–October 2017) follow-up. The effect of the intervention on client-reported receipt of preventive care (assessment, advice, and offer and acceptance of referrals) for risk behaviours was reported separately [50]. The Human Research Ethics Committees of Hunter New England Health (Ref no. 16/02/17/4.09) and the University of Newcastle (Ref no. H-2016-0123) approved the research. The trial was prospectively registered on the Australian New Zealand Clinical Trials Registry (ACTRN12616001519448). The study adhered to the Consolidated Standards of Reporting Trials (CONSORT) guidelines (Additional File 1 contains the CONORT checklist [51]).

### Economic study design

A within-trial evaluation of cost and cost-effectiveness was retrospectively undertaken, adopting the cost perspective of the mental health service in order to capture the direct costs incurred by the mental health service to implement the model of preventive care. The outcome of the economic analysis was the acceptance of referrals to the free telephone coaching services: NSW Get Healthy service and the NSW Quitline. The selection of this outcome aligns with the local health district’s policy. Therefore, reporting this outcome in the economic evaluation directly informs decision-makers about the cost of adhering to this chronic disease prevention policy. Furthermore, of the outcomes examined in the effectiveness analysis, this outcome was the most pertinent outcome captured by the trial as it is the outcome that the mental health service can directly influence. Moreover, research indicates that providing a referral may be the most effective element of preventive care for producing long-term behaviour change [18, 52]. The time horizon was the 1-month follow-up of the trial. The economic evaluation adhered to the Consolidated Health Economic Evaluation Reporting Standards (CHEERS) checklist (Additional File 2 contains CHEERS checklist [53]).

### Intervention

A specialist preventive care clinician (an occupational therapist) who had the dedicated role of offering and providing clients an additional consultation (approximately 40-min) and follow-up was embedded in the community mental health service for six-months. The objective of the position was to provide preventive care (assessment, advice, and referral) and to motivate clients to accept referrals to existing free telephone coaching services. The clinician telephoned clients approximately two-weeks following their consultation to follow-up on their progress and offer any additional advice or assistance necessary.

### Trial data collection procedures and measures

Eligible clients were mailed an information statement and provided an opportunity to opt-out of the study. All remaining clients were contacted by trained telephone interviewers at baseline and follow-up to obtain verbal consent to participate and collect the following information via computer assisted telephone interviews (CATI; questions developed based on the research team’s previous research in this area [31, 54]):

1. Engagement in health risk behaviours in the previous month, being: current tobacco smoking status, daily serves of fruit and vegetable consumed, consumption of alcohol, and minutes of physical activity a week (walking, moderate, and vigorous activity).
2. If participants had been offered a referral to a relevant telephone service from the mental health service (“did the mental health service offer to arrange for the Get Healthy Service/Quitline to call you?”), and if so, if they accepted (“did you accept this offer?”). There was no distinction as to whether the referral was offered by their usual treating clinician or the specialist clinician.

Data from participants who were eligible for a referral (i.e. not meeting Australian National guidelines for the risk behaviours [55–58]; Table 1) were used to calculate: [1] acceptance of a referral to the Get Healthy Service (of participants who were not meeting guidelines for nutrition, alcohol, and/or physical activity; yes vs no/don’t know/referral not offered) and [2] acceptance of a referral to the Quitline (of participants who were currently smoking; yes vs no/don’t know/referral not offered).

**Table 1:** Definitions of eligibility for a referral

Telephone coaching service	Eligibility for a referral (not meeting Australian National guidelines)
The Get Healthy Information and Coaching Service	At least one of the following:
	1. eating less than two serves of fruit or fives serves of vegetables [55];
	2. consuming more than 2 standard drinks on an average day or more than 4 standard drinks on any one occasion [56]; or
	3. engaging in less than 150 min of moderate intensity physical activity or 75 min of vigorous intensity physical activity, or any equivalence combination of each, weekly [57].
The Quitline	Currently smoking [58].

### C**ost data collection methods and measures**

#### Literature review

A literature review of similar studies was undertaken to ensure all pertinent costs were considered for costing the model of care, with a focus on studies that contained an economic evaluation of a dedicated clinician(s) providing preventive care (for any component of physical health) in any health setting. The following databases were searched from database inception to 10/01/2018: Embase, Medline, PsycINFO, and Cochrane Central Register of Controlled Trials (CENTRAL; see Additional File 3 for search terms). The review indicated that the following costs should be considered: salary for the specialist clinician [59], room space/premises costs for the specialist clinician [59], administrative costs [60], travel costs for client home visits (where required) [61, 62], printing of any supporting materials [59], training costs for the specialist clinician [44, 59], and telephone calls (made by the specialist clinician to arrange consultations and/or follow-up on client progress) [59, 61].

#### Data collection and measures

A retrospective economic analysis was undertaken to determine the incremental cost of the model of care in a community mental health service. Costs are reported in 2018 Australian dollars. Informed by the literature review, all contributing costs were identified, collected, and aggregated. The specialist preventive care clinician wage was determined by the relevant award (level 3, year 2 health professional) [63]. The specialist clinician recorded their weekly activities and estimated their time engaged in these activities each week (see Additional File 4). Only time engaged in clinical tasks, relevant to the role of specialist preventive care clinician, were included in the costing analysis so as to determine the cost-effectiveness of the model of care in an applied service context [64].

A bottom-up micro-costing approach guided the collection of all itemised costs [64]. The wage for reception staff was determined by the relevant award (level 1, year 3) [65]. The community mental health service provided an estimate for the time required of reception staff to support the intervention by receiving and directing clients visiting the service, and answering incoming calls to the clinician. The costs of telephoning clients (to arrange consultations and deliver follow-up calls) were based on published costs of the telephone provider [66]; printing costs (paper and ink) were accessed from an office supplies store [67–69]; and travel costs were based on standard cents/km rates from the Australian Tax Office [70]. Overheads were calculated as 27.5% of the cost of labour, based on the standard factor for the health district [71]. The mental health service estimated the room space required to hold the additional consultation (12m^2^), and the cost of this space was determined on an average for the local area [72].

For both the intervention and usual care conditions, it was assumed that no additional costs were incurred for the delivery of usual preventive care in routine mental health consultations.

### Analytical methods

All analyses were undertaken using Microsoft Excel Software, 2013.

#### Cost

A cost model was constructed in a Microsoft Excel workbook where costs were entered and analysed.

#### Base case cost-effectiveness analysis

Two separate analyses were undertaken to determine the cost-effectiveness of the model of care in increasing client acceptance of referrals to each telephone service, for participants eligible to receive each referral. Costs were apportioned according to the eligibility for a referral to each telephone service among participants (see Additional file 5). Additionally, to determine the cost-effectiveness of the total intervention across both outcomes and for the whole sample (regardless of referral eligibility), cost-effectiveness was also calculated for referral acceptances to both the Get Healthy Service and Quitline combined.

A complete case analysis was performed. Total costs were adjusted to account for missing data. Intervention delivery and participation in data collection were independent, such that clients could receive the intervention (i.e. incur a cost) and not participate in data collection. Therefore, to estimate the appropriate cost of the intervention attributable to participants who provided data, the total cost of intervention delivery was firstly calculated. Data were missing for 54% of intervention participants at the follow-up. The total intervention cost was adjusted accordingly to estimate the proportion of intervention costs attributable to the participants with available data, where costs were reduced to 46% of the total amount.

The excel workbook was programmed to undertake 1000 bootstrapped iterations [64]. The median incremental cost-effectiveness ratio (ICER) was taken from these iterations and expressed as cost per percentage point increase in the acceptance of referrals from the mental health service to the i) Get Healthy Service, ii) Quitline, and iii) both telephone services combined. Bootstrapping was used to determine 95% confidence intervals [64]. The ICERs were plotted to produce cost-effectiveness planes, graphing the cost compared to effectiveness of the model of care, relative to usual service delivery. Lastly, cost-effectiveness acceptability curves were constructed to quantify the probability of the intervention being cost-effective for each of the outcomes, at varying levels of willingness to pay [64]. A Bayesian adjustment was undertaken using R statistical software for one outcome, acceptance of referral to the Quitline, due to the number of events/non-events being zero [73]. The beta posterior distributions used for simulation were based on conjugate binomial likelihoods and a uniform prior [73].

#### Sensitivity analysis

Univariate sensitivity analyses tested the effect of plausible variations in key input parameters on the ICERs. Firstly, a number of studies have explored the potential of nurse-led interventions for promoting risk behaviour change among people with a mental illness [38, 74, 75]. Therefore, a sensitivity analysis was undertaken using a lower resource cost, where the specialist preventive care clinician salary was based on the public health system nurses’ and midwives’ state award (4th year registered nurse) [76].

Secondly, research has suggested that receiving healthy lifestyle support from a peer worker (defined by the NSW Mental Health Commission as a person with a lived experience of mental illness in addition to the skills and qualifications required to undertake a specific role [77]) may be acceptable [78] and that the potential of a peer worked in this role should be explored. Based on recommendations from the service, this salary was taken from the NSW health service health professionals award [63].

Thirdly, the involvement of receptionists may vary between services, where a service may have receptionists more heavily involved in performing non-clinical tasks (e.g. arranging consultations and faxing referral forms) and less clinician time to do so. A sensitivity analysis explored a scenario where there was an increase in the time allocated to reception staff support (additional 5 hours per week), and a decrease in the time allocated to the specialist clinician (reduction of 5 hours per week). This time estimate was determined based on process data collected by the specialist clinician (see Additional File 4).

Lastly, increasing the effectiveness of the intervention on client uptake (20% increase in referral acceptances) was tested.

#### Budget impact analysis: managerial perspective

A budget impact analysis (BIA) determines the expected financial or accounting impact of implementing an intervention [45, 46]. This analysis takes a managerial perspective and provides a basis for decision makers in health services to assess the affordability of a new intervention, given the practical constraints of available health care funding and other potential uses of that funding [79]. Informed by the CEA, a BIA was undertaken in accordance with a published checklist developed in accordance with the International Society for Pharmacoeconomics and Outcomes Research (ISPOR) recommendations [45]. The aim was to project the costs of implementing and sustaining the specialist preventive care clinician over a five-year time horizon for the whole client population of the mental health service, coupled with the projected effect in terms of number of referral acceptances (to the Quitline, Get Healthy Service, and total referral acceptances combined). The BIA considers costs for all prospective clients of the service.

A prevalence cohort model was used [45]. The number of annual clients was provided by the mental health service. The uptake of the intervention and direct effects were estimated using trial data. The cost of implementing the intervention for the total client population was calculated using identical methods to the base case analysis. A sensitivity analysis estimated budget impact if a registered nurse was employed in the specialist clinician role [76].

## Results

### Sample characteristics

Figure 1 displays participant flow through the trial. At baseline, 389 clients completed or partially completed the interview (intervention *n* = 203, usual care *n* = 186), and 367 completed or partially completed the follow-up interview (intervention *n* = 186, control *n* = 181). Chi-square tests indicated that the completion rates at baseline and follow-up did not significantly differ by condition (χ2(1) = .698, *p* = 0.193; and χ2(1) = 0.121, *p* = 0.728, respectively). Characteristics of the sample are presented in Table 2. There were no significant differences between the groups at baseline or follow-up in terms of clinical and demographic characteristics (assessed using chi-square tests for categorical and t-tests for continuous variables).

**Table 2:** Characteristics of the sample

Outcomes	Baseline		Follow-up	
	Intervention	Usual care	Intervention	Usual care
	(*n* = 203)	(*n* = 186)	(*n* = 186)	(*n* = 181)
	% (n)	% (n)	% (n)	% (n)
Gender (%)				
Male	54.2 (110)	52.2 (97)	54.3 (101)	52.5 (95)
Female	45.8 (93)	47.8 (89)	45.7 (85)	47.5 (86)
Age (years)				
Mean (SD)	40.6 (12.9)	39.7 (13.0)	41.2 (12.9)	40.2 (12.7)
Median (range)	40 (18–66)	39 (18–66)	41 (18–66)	40 (18–66)
Diagnosis Type (%)				
Psychotic/Schizophrenia	36.5 (74)	41.4 (77)	34.9 (65)	40.9 (74)
Mood Disorders	37.4 (76)	33.9 (63)	39.2 (73)	34.3 (62)
Anxiety and Stress Related Disorders	15.8 (32)	8.2 (24)	16.1 (30)	12.7 (23)
Other	10.3 (21)	11.8 (22)	9.7 (18)	12.2 (22)
Relationship status (%)				
Single	58.4 (118)	63.4 (118)	56.8 (105)	61.3 (111)
Married/De facto	21.8 (44)	15.6 (29)	21.1 (39)	16.6 (30)
Separated/Divorced/Widowed	19.8 (40)	21.0 (39)	22.2 (41)	22.1 (40)
Identified as Aboriginal and/or Torres Strait Islander (%)				
Yes	11.9 (24)	10.3 (19)	14.1 (26)	8.3 (15)
No	88.1 (178)	89.97(166)	85.9 (159)	91.7 (165)
Employment Status (%)				
Full time	10.9 (22)	5.9 (11)	11.4 (21)	5.5 (10)
Part time or casual	12.9 (26)	14.0 (26)	16.8 (31)	13.8 (25)
Household Duties/Student	32.7 (66)	49.2 (73)	31.4 (58)	36.5 (66)
Unemployed	34.2 (69)	33.3 (62)	32.4 (60)	37.6 (68)
Retired	4.5 (9)	4.8 (9)	3.8 (7)	5.0 (9)
Other	5.0 (10)	2.7 (5)	4.3 (8)	1.7 (3)
Highest education level achieved (%)				
Less than school certificate	16.8 (34)	15.1 (28)	18.4 (34)	13.3 (24)
School certificate	22.3 (47)	25.8 (48)	22.7 (42)	25.4 (46)
Higher school certificate	18.8 (38)	15.1 (28)	15.7 (29)	16.0 (29)
TAFE or Diploma	29.7 (60)	34.9 (65)	30.3 (56)	36.5 (66)
Bachelor/Post Graduate Degree	11.4 (23)	9.1 (17)	13.0 (24)	8.8 (16)
Length of treatment for the current episode of care (months)				
Mean (SD)	31.5 (54.8)	25.1 (46.1)	33.6 (57.0)	27.6 (49.7)
Median (range)	8 (1–258)	7 (1–235)	8 (1–258)	7 (1–235)
Eligible for a referral				
Quitline: current tobacco smoking	48.5 (98/202)	50.8 (94/185)	47.3 (88/186)	55.8 (101/181)
Get Healthy: at least one of poor nutrition, harmful alcohol consumption, and/or physical inactivity	94.1 (190/202)	96.2 (179/186)	95.2 (177/186)	96.1 (174/181)

*Note.* Ns vary due to missing data; *SD* standard deviation; *TAFE* Technical and Further Education

### Trial outcomes

From baseline to follow-up, there was a significantly greater absolute increase in the proportion of intervention participants accepting referrals to the Get Healthy Service (0.5% at baseline to 19.2% at follow-up; absolute increase + 18.7%) and the Quitline (1.0 to 11.4%; + 10.4% increase), as compared to usual care (Get Healthy Service: 1.1 to 0.6%; − 0.5% decrease; Quitline: 1.1 to 0%; − 1.1% decrease, respectively). The change between time points was 93.3 (risk ratio; 95% CI: 6.5–1341.2, *p* < .001) and 19.8 (risk ratio; 95% CI: 1.0–952.0, *p* = .040) times greater in the intervention compared to usual care group for acceptance of referrals to the Get Healthy Service and Quitline, respectively.

### Intervention costs

Table 3 shows the breakdown of intervention costs. The total cost of embedding the specialist preventive care clinician in the community mental health service for 6 months was $27,684.41, being $68.19 per intervention participant.

**Table 3:** Itemised costs of embedding specialist preventive care clinician in the community mental health service for 6 months

	Cost per unit	Unit	Units utilised (n)	Totals	Adjusted^1^ totals ($)	Reference
**Base case intervention costs**						
Labour						
Specialist clinician training (occupational therapist)	$49.69	Hours	16	$794.97	$794.97	[63]
Specialist clinician salary (occupational therapist)	$49.69	Hours	355.2	$17,648.34	$8085.20	[63]
Reception staff support	$24.56	Hours	8	$196.44	$90.00	[65]
Premises cost/room space	$245.00	Metres^2^	12	$2940.00	$2940.00	[72]
Telephone calls	$560.00	Phone plan	1	$560.00	$256.55	[66]
Printing						
Self-help pamphlets	$0.43^2^	Pamphlets	100	$69.00	$31.61	[67, 68]
Fax referral forms	$0.23^3^	Referrals	84	$41.16	$18.86	[67, 69]
Travel costs	$0.66	km	540	$356.40	$163.28	[70]
Overheads (% of total labour)	27.5%	%		$5125.93	$2466.00	[71]
Total base case intervention Costs				$27,684.41	$14,825.34	
Base case cost per participant				$68.19		
**Parameters varied in sensitivity analyses (all non-specified parameters remain the same as the base case)**						
**(i) Registered nurse**						
Specialist clinician training (registered nurse)	$38.86	Hours	16	$590	$590	[76]
Specialist clinician salary (registered nurse)	$38.86	Hours	355.2	$13,092	$5998	[76]
Total intervention costs sensitivity analysis (i)				$21,613	$11,902	
**(ii) Peer worker**						
specialist clinician training (peer worker)	$32.81	Hours	16	$525	$525	[63]
Specialist clinician salary (peer worker)	$32.81	Hours	355.2	$11,656	$5955	[63]
Total intervention costs sensitivity analysis (ii)				$19,699	$10,981	
**(iii) Reception support increased**						
Specialist clinician salary	$49.69	Hours	235.2	$11,686	$5354	[63]
Reception staff support	$24.56	Hours	128	$3143	$1440	[65]
Total intervention costs sensitivity analysis (iii)				$23,839	$13,064	

^1^The cost of the intervention for participants with available data. Total intervention cost was adjusted to estimate the proportion of intervention costs attributable to participants with available data, where costs were reduced to 46% of the total amount

^2^Printed in colour

^3^Printed in black and white

### Base case

**Fig. 2:**
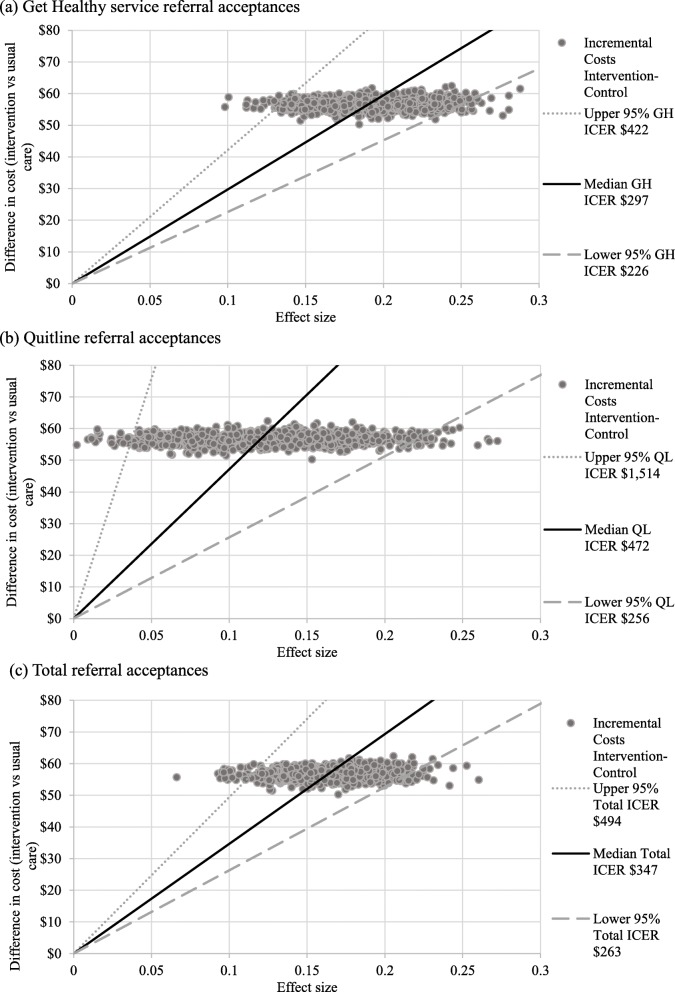
Cost-effectiveness planes

Incremental cost-effectiveness ratios (ICERs) represent the incremental cost per additional referral acceptance in the intervention, compared to usual care group. The ICERs were: $287 per additional Get Healthy Service referral acceptance (95% CI: $226–$422), $472 per additional Quitline referral acceptance (95% CI: $256–$1514), and $347 per total referrals acceptances to both services combined (95% CI: $263–$494). Analysis of the bootstrapped ICERs found that 100% of cases were in the north-east quadrant for all three outcomes, indicating that the intervention was both more costly and more effective than usual care [64] (Fig. 2).

Cost-effectiveness acceptability curves indicated that the incremental costs ranged from $264–$372 per Get Healthy Service referral acceptance, $348–$980 per Quitline referral acceptance, and $306–$432 per total referral acceptances combined, from a 20 to 90% probability of cost-effectiveness, respectively (see Additional File 6).

### Sensitivity analysis

**Fig. 3:**
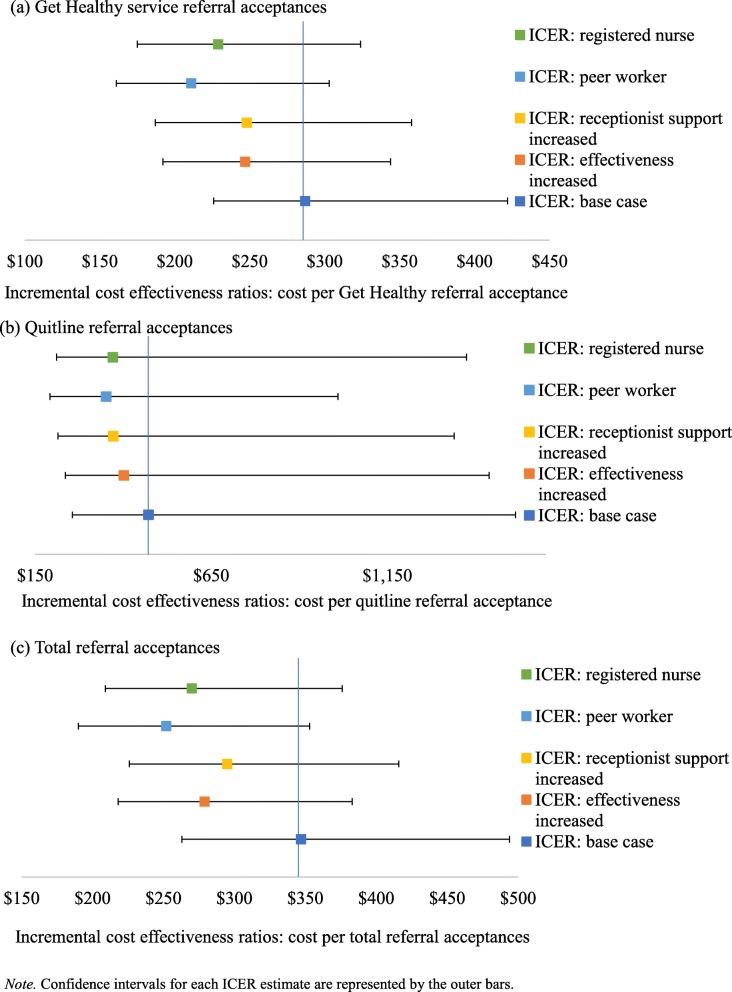
ICERs and confidence intervals for sensitivity analyses. *Note.* Confidence intervals for each ICER estimate are represented by the outer bars.

Figure 3 outlines the results of the sensitivity analysis. Employing a registered nurse in the role (Test i) resulted in lower ICERs: $229 ($175–$324) for the Get Healthy Service, $371 ($211–$1375) for the Quitline, and $270 ($209–$376) for both telephone services combined. A peer worker in this role (Test ii) also lowered the ICERs: $211 ($161–$303) for the Get Healthy Service, $352 ($192–$1010) for the Quitline, and $252 ($190–$353) for both telephone services combined. Test (iii) increased the involvement of reception staff, yielding lower ICERs: $248 ($187–$358) for the Get Healthy Service, $372 ($215–$1340) for the Quitline, and $295 ($226–$4166) for both telephone services combined. Test (iv) increased intervention effectiveness by 20%, resulting lower ICERs: $247 ($192–$344) for the Get Healthy Service, $402 ($236–$1439) for the Quitline, and $279 ($218–$383) for both telephone services combined.

### Budget impact analysis

The budget impact analysis is presented in Table 4. The prospective client population (i.e. the total number of eligible clients of the community mental health service) was estimated to be 8927 over the 5 years. The projected cost to upscale and implement the model of care for the whole client population for a period of 5-years was $711,446. The annual cost was $137,393 in the first year (2018), resulting in an expected impact of 382 annual acceptances of referrals across telephone services (87 to the Quitline and 295 to the Get Healthy service). Annual costs were projected to increase each year, being up to $148,060 in the fifth year (2022). Sensitivity analysis indicated that if a registered nurse was in the position, the total cost was decreased to $540,559 (annual cost ranging between $104,410 in the first year to $112,502) in the fifth year).

**Table 4:** Budget impact analysis

1. Time Line^1^	2018	2019	2020	2021	2022
Eligible Clients (n)^2^	1616	1696	1781	1870	1964
Clients Accessing Intervention (n)	441	487	537	592	652
Intervention Access Rate (a/g)	27.3%	28.7%	30.1%	31.6%	33.2%
2. Intervention Costs	($)	($)	($)	($)	($)
Clinical					
Clinician’s Counselling Labour	$98,516	$100,585	$102,697	$104,854	$107,056
Overheads (27.5% of labour cost)	$27,092	$27,661	$28,242	$28,835	$29,440
Implementation					
Clinician’s Training Labour	$795	$0	$0	$0	$0
Administrative Labour	$782	$821	$862	$905	$950
Premises cost/room space	$5880	$5880	$5880	$5880	$5880
Telephone calls	$2229	$2340	$2457	$2580	$2709
Printing	$248	$260	$273	$287	$301
Clinician Travel	$1418	$1489	$1564	$1642	$1724
Overheads (27.5% of labour cost)	$434	$226	$237	$249	$261
Total (Incremental Cost)^3^	$137,393	$139,036	$141,975	$144,982	$148,060
Cost of Intervention per Client	$85.04	$81.96	$79.70	$77.52	$75.39
3. Net Impacts	(n)	(n)	(n)	(n)	(n)
Expected Incremental Referrals Acceptances^4^					
Quitline	87	101	116	135	156
Get Healthy service	295	342	396	458	530
Total referral acceptances	382	442	512	593	686

^1^An annual increase for the following parameters was assumed: 5% annual increase in client population, 5% annual increase in uptake of the preventive care consultation, 2.1% annual increase in the salary of the specialist clinician, and a 5% annual increase in the incremental effectiveness of the intervention. In line with these increases, an incremental annual increase in implementation costs (administrative, telephone calls, printing and travel) was calculated accordingly;

^2^Represents the number of clients estimated to be eligible to attend the additional consultation i.e. excluding those identified as too unwell (based on trial data);

^3^The intervention is an add on to Usual Care, hence all its costs are the incremental costs;

^4^Based on percentages (effect/population) in study

## Discussion

This is the first Australian study to examine the cost-efficiency and affordability of embedding a specialist preventive care clinician in a community mental health service to enhance the universal provision of care to reduce preventable chronic disease risk behaviours. This applied economic analysis determined the cost for the mental health service to adhere to key policy targets, with the cost of the model of care being $68.19 per client randomised to the intervention condition (*n* = 406). The BIA estimated that the costs to implement the model of care for all prospective clients of the community mental health service was $711,446 over 5-years (*N* = 8927 clients), generating 2616 accepted referrals. The findings suggest that the model of care involves a relatively low per-client cost [59, 61] and demonstrates the cost to achieve guideline and policy-concordant preventive care delivery in a mental health service. As such, this information is directly relevant to decision makers in health services.

This study is one of few economic evaluations of interventions that address the poor physical health of people with a mental illness [80, 81], and one of only two studies that have examined the cost-effectiveness of a specialist preventive care clinician in a community mental health service [44] Although similar ICERs were not available for the present findings to be compared to previous cost-effectiveness studies, the cost per-participant results for the present study are comparable to previous studies reporting the cost-effectiveness of specialist clinicians in general health settings [59, 61].

The largest cost of the intervention was attributable to the labour of the clinician, accounting for approximately 60% of the total intervention costs. In the present study, the specialist clinician’s clinical workload was not equivalent to a full-time position. This may be contributed to by the intervention uptake rate, where less than half of intervention participants took up the additional consultation. Depending on the size and location of individual mental health services, there is potential that one full-time equivalent clinician could treat clients across multiple services, or only a part-time position may be required for smaller services; thereby improving cost-effectiveness by maximising the number of clients utilising the specialist clinician’s services. Future research may determine the critical client population size per service to avoid saturation of the clinician’s capacity, whilst maximising the number of clients utilising their services in order to optimise cost-effectiveness. A job evaluation and classification analysis for the institution of this new role may objectively determine the time, qualifications, skills and experience required to fulfil the position’s responsibilities [82].

Sensitivity analyses were undertaken to further explore the results under different, plausible assumptions. Lower ICERs compared to the base case analysis were observed across outcomes for all sensitivity analyses, demonstrating the potential to improve cost-effectiveness in future research and implementation of service delivery models. These parameters should be considered by policy makers and service providers in the context of the specific circumstances of different services and health districts. Across all outcomes, the lowest ICERs were found when testing a peer-worker employed as the specialist preventive care clinician. However, the sensitivity analyses varying the health professional employed as the specialist clinician are constrained as they did not consider any variation in effectiveness. For example, previous research indicates that behaviour change interventions may be perceived as more acceptable when being provided by a peer than a mental health clinician [78], however, their relative effectiveness has not been explored. Future research should consider the potential of a peer worker in this role.

The budget impact analysis estimated the resources required to implement the model of care for the whole client population of the community mental health service. In contrast with previous research conducted in the US [44], the model of care tested in the present study did not attract revenues. The resources required to fund the implementation of the model of care were projected to increase annually (in line with projected increases in the client population and labour costs), ranging from $137,393 in the first to $148,060 in the fifth year. This budget impact statement is expected to be of key interest to decision makers, providing a projection of the costs of implementing the model of care to meet the key policy target of referral to specialist chronic disease prevention services. Due to the use of trial-based evidence for the budget impact statement, it did not calculate savings for the health system based on reduced use of downstream healthcare resources as a consequence of exposure to the new model of care.

The study results should be considered in the context of its limitations. Firstly, a key limitation of the present study was the consideration of only immediate (within-trial) costs and benefits to the mental health service; without consideration of downstream and long-term costs. Given this was a retrospective, trial-based analysis, downstream costs and costs avoided were not available. Hence, modelling of such costs without a good evidence-base would introduce considerable, and unpredictable, bias. While the within trial analysis provides useful information to decision makers on the cost-efficiency and affordability of implementing the model of care in the mental health service, further research is needed to explore cost-effectiveness with a broader, societal perspective and a longer time horizon. This future research should consider the complexity of public health interventions, and could use a systems approach to the larger-scale design and economic evaluation of the specialist clinician model [83]. Furthermore, modelling patient outcomes such as quality-adjusted life years was not logistically possible within the present study, but would be preferable to provide a more comprehensive estimate of the costs and benefits of the model of care and further inform service delivery planning [84]. Secondly, the intervention was implemented at a single mental health service and in the context of policies specific to the state and health district. Generalisability to other contexts is constrained as other services may have different preventive care policies and key performance indicators. Thirdly, primary outcome data for the trial as described in the study protocol (client referral to and uptake of the telephone services as obtained from the respective services) were not available. Client-reported acceptance of referrals was therefore chosen as the most pertinent outcome to enable examination of costs; relevant to state and district wide policies and the service’s key performance indicators. Client self-report has been demonstrated to be an acceptable measure of receipt of preventive care [85, 86]. Lastly, as the model of care increased the number of accepted referrals to specialist telephone services, its wider implementation would need to occur in consultation with each telephone service to determine the ability of the services to support the increased number of clients, for example, whether their workforce could meet this demand.

A strength of the present study includes the diagnostically heterogeneous sample, which has been recognised as important given that previous economic evaluations have largely focused on specific diagnoses or categories, limiting generalisability [80]. Secondly, the present study examined an intervention that simultaneously targeted multiple risk behaviours. Such multi-risk interventions have been suggested to be more efficient and cost-effective than interventions targeting only one risk [87, 88], given that they provide ability to address multiple risks at a time for a similar start-up and delivery cost. Lastly, this applied economic evaluation provides a comprehensive assessment of the cost-efficiency and affordability of meeting clinical practice guideline and policy targets for mental health services. Such an assessment forms an important step in preparing a Business Case, which would provide robust evidence to support evidence-based decisions regarding resource allocation [89].

## Conclusions

The specialist clinician model of care significantly increased the number of clients of a community mental health service accepting referrals to telephone behaviour change services, at a cost of $68 per participant. Given that referral to specialist services is a key pillar of chronic disease prevention policy, the results of this study will inform decision-makers regarding the cost for a mental health service to adhere to such clinical practice guidelines and policies, and deliver evidence-based preventive care to people with a mental illness. Future research should undertake modelling to determine the economic impact of the model of care when considering client outcomes in a cost-utility study, and taking a broader societal perspective considering downstream and long-term costs.

## Supplementary information

**Additional file 1.** Populated CONSORT 2010 checklist.

**Additional file 2.** Populated CHEERS checklist.

**Additional file 3.** Search strategy for literature review.

**Additional file 4.** Breakdown of the specialist preventive care clinician’s activities.

**Additional file 5.** Extended explanation of apportioning of costs.

**Additional file 6.** Cost-effectiveness acceptability curves.

## Data Availability

The datasets generated and analysed during the current study are not publicly available to preserve the privacy of participants; however, they are available from the corresponding author on reasonable request.

## Abbreviations

CATI: Computer assisted telephone interviews
ICER: Incremental cost-effectiveness ratio
NSW: New South Wales
BIA: Budget impact analysis
IPSOR: International Society for Pharmacoeconomics and Outcomes Research
CHEERS: Consolidated Health Economic Evaluation Reporting Standards
CONSORT: Consolidated Standards of Reporting Trials
SD: Standard deviation
TAFE: Technical and Further Education
