# Supplementary material for: An economic evaluation of a specialist preventive care clinician in a community mental health service: a randomised controlled trial
Source: BMC Health Serv Res. 2020 May 11;20:405. doi: 10.1186/s12913-020-05204-7 (PMC7212584; doi:10.1186/s12913-020-05204-7)
Supplement: Supplementary file 3 — Additional file 3. Search strategy for literature review. [file 12913_2020_5204_MOESM3_ESM.docx]

**Additional File 3: search strategy for literature review**

**Search strategy for Embase, Medline and PsycINFO**

Database(s): **Embase**1947 to present**, Medline**1946 - present**, PsycINFO**1806 to January Week 1 2018 
Search Strategy:

| **#** | **Searches** | **Results** |
| --- | --- | --- |
| 1 | health facilities/ or hospitals/ or health services/ or community health services/ or mental health services/ or community mental health services/ or social work, psychiatric/ or preventive health services/ | 879934 |
| 2 | Hospitals, Psychiatric/ | 56600 |
| 3 | inpatients/ or outpatients/ | 215313 |
| 4 | ((psychiatric or mental health or health) adj3 (centre* or center* or service* or hospital* or clinic* or ogani?ation*)).tw. | 611349 |
| 5 | Family Practice/ or General Practice/ or Primary Health Care/ | 287418 |
| 6 | 1 or 2 or 3 or 4 or 5 | 1783935 |
| 7 | Smoking/ or Smoking Cessation/ | 472053 |
| 8 | smoking.tw. | 525361 |
| 9 | nutrition*.mp. | 847982 |
| 10 | Fruit/ | 99545 |
| 11 | Vegetables/ | 58554 |
| 12 | Diet/ | 383993 |
| 13 | (fruit* or vegetables* or diet*).tw. | 1401006 |
| 14 | drinking behaviour/ or alcohol drinking/ | 109891 |
| 15 | binge drinking/ | 6805 |
| 16 | (drinking or alcohol*).tw. | 1006299 |
| 17 | exercise/ | 377572 |
| 18 | physical fitness/ | 64818 |
| 19 | sedentary lifestyle/ | 17328 |
| 20 | (physical activit* or exercise or physical fitness or physical inactivit* or sedentary).tw. | 820498 |
| 21 | (lifestyle* adj3 (behavior* or behaviour*)).tw. | 12398 |
| 22 | (risk adj3 (behavior* or behaviour*)).tw. | 100441 |
| 23 | 7 or 8 or 9 or 10 or 11 or 12 or 13 or 14 or 15 or 16 or 17 or 18 or 19 or 20 or 21 or 22 | 4405752 |
| 24 | (specialist or ((addition* or extra or increase* or new or specific) adj3 (staff* or clinician* or role* or personnel or practitioner))).tw. | 304774 |
| 25 | Cost*.ti. | 263707 |
| 26 | Cost*.ab. | 1210296 |
| 27 | economic.ti. | 87533 |
| 28 | economic.ab. | 452987 |
| 29 | 25 or 26 or 27 or 28 | 1658910 |
| 30 | 6 and 23 and 24 and 29 | 558 |
| 31 | limit 30 to (yr="2000 -Current" and english) | 477 |

**Search strategy for the Cochrane Central Register of Controlled Trials (CENTRAL)**

((psychiatric or mental health or health) near3 (centre* or center* or service* or hospital* or clinic* or ogani?ation*)) or inpatient* or outpatient* or psychiatric hospital* or family practice or general practice or (primary near2 care)

and

tobacco or smoking or smoking cessation or nutrition or fruit* or vegetable* or diet or alcohol or alcohol drinking or binge drinking or exercise or physical fitness or sedentary lifestyle or physical activit* or physical inactivit* or (lifestyle near3 (behaviour* or behaviour*)) or (risk near3 (behaviour* or behavior*))

and

(specialist or ((addition* or extra or increase* or new or specific) near3 (staff* or clinician* or role* or personnel or practitioner)))

and

cost* or economic (title and abstract)

2000 onwards

496 hits
