# Supplementary material for: An economic evaluation of a specialist preventive care clinician in a community mental health service: a randomised controlled trial
Source: BMC Health Serv Res. 2020 May 11;20:405. doi: 10.1186/s12913-020-05204-7 (PMC7212584; doi:10.1186/s12913-020-05204-7)
Supplement: Supplementary file 4 — Additional file 4. Breakdown of the specialist preventive care clinician’s activities. [file 12913_2020_5204_MOESM4_ESM.docx]

**Additional file 4.** Breakdown of the specialist preventive care clinician’s activities.

| **Table A4.1.** Breakdown of the specialist preventive care clinician’s time | | |
| --- | --- | --- |
| **Task description** | **Hours/week** | **(%)** |
| 1. **Research tasks** |  |  |
| Generating lists of current clients and entering details into research database | 8 | 22.0% |
| Weekly meetings and phone calls with researchers | 2 | 5.5% |
| Updating and managing intervention delivery database | 5 | 13.8% |
| Total research hours | 15 | 41.3% |
| 1. **Miscellaneous other tasks** |  |  |
| Providing supervision to new staff | 1 | 2.8% |
| Receiving supervision | 0.5 | 1.4% |
| Attending meetings | 2 | 5.5% |
| Attending workshops and receiving training | 3 | 8.3% |
| Total miscellaneous hours | 6.5 | 17.9% |
| 1. **Clinical tasks for healthy lifestyle intervention** |  |  |
| Phoning clients to arrange appointments | 4 | 11.0% |
| Holding appointments^2^ | 3.3 | 9.1% |
| Follow-up calls | 2.5 | 6.9% |
| Administrative tasks (e.g. making clinical notes, faxing referrals) | 3 | 8.3% |
| Travel time (for appointments held off-site) | 2 | 5.5% |
| Total clinical hours | 14.8 | 40.8% |
| **Total hours per week** | 36.3 | 100.0% |
