# Supplementary material for: An economic evaluation of a specialist preventive care clinician in a community mental health service: a randomised controlled trial
Source: BMC Health Serv Res. 2020 May 11;20:405. doi: 10.1186/s12913-020-05204-7 (PMC7212584; doi:10.1186/s12913-020-05204-7)
Supplement: Supplementary file 5 — Additional file 5. Extended explanation of apportioning of costs. [file 12913_2020_5204_MOESM5_ESM.docx]

**Additional file 5: extended explanation of apportioning of costs**

Costs were allocated across the entire intervention group, according to the proportion of eligibilities in the sample (table A5.1). As the specific purpose of the intervention was to encourage acceptance of referrals to the Get Healthy service and Quitline, costs were allocated according to the proportion of the referral eligibilities among participants. However, clients who were not eligible for either service are not represented in these outcomes, and may still attend the additional preventive care consultation, thereby incurring a cost. Therefore, an outcome reflecting the total number of referral acceptances (to either service) was calculated for the entire intervention group (considering the total intervention cost).

**Table A5.1.** Allocation of intervention costs across referral eligibilities

| 1. **Participants** | **n** |
| --- | --- |
| Available data at follow-up | 220 |
| Data missing at follow-up | 186 |
| 1. **Intervention costs** | **$** |
| Unadjusted total intervention costs | $27,684 |
| Adjusted total intervention costs (for missing values) | $14,825^1^ |
| 1. **Eligibility for a referral** | **n** |
| Across participants |  |
| Quitline | 2 |
| Quitline and Get Healthy | 86 |
| Get Healthy only | 91 |
| Neither service | 7 |
| Across eligibilities |  |
| Quitline | 88 (32.4%) |
| Get Healthy | 177 (65.1%) |
| Neither service | 7 (2.6%) |
| Total eligibilities | 272^2^ |
| 1. **Cost across eligibilities** | **$** |
| Cost per eligibility (total eligibilities/adjusted total intervention costs) | $54.50 |
| Allocated cost: Quitline | $4,796.43 (32.4%)^3^ |
| Allocated cost: Get Healthy | $9,647.37 (65.1%)^4^ |
| Allocated cost: neither service | $381.53 (2.6%) |

^1^Cost is considered for the outcome: total referral acceptances.
^2^represents the total number of eligibilities in the sample (not the number of participants with available data, as participants could be eligible for more than one service).
^3^Cost is considered for the outcome: Quitline referral acceptances.
^4^Cost is considered for the outcome: Get Healthy service referral acceptances.
