# Supplementary material for: An economic evaluation of a specialist preventive care clinician in a community mental health service: a randomised controlled trial
Source: BMC Health Serv Res. 2020 May 11;20:405. doi: 10.1186/s12913-020-05204-7 (PMC7212584; doi:10.1186/s12913-020-05204-7)
Supplement: Supplementary file 6 — Additional file 6. Cost-effectiveness acceptability curves. [file 12913_2020_5204_MOESM6_ESM.docx]

**Additional file 6: cost-effectiveness acceptability curves**

**Figure A6.1.** Cost-effectiveness acceptability curves


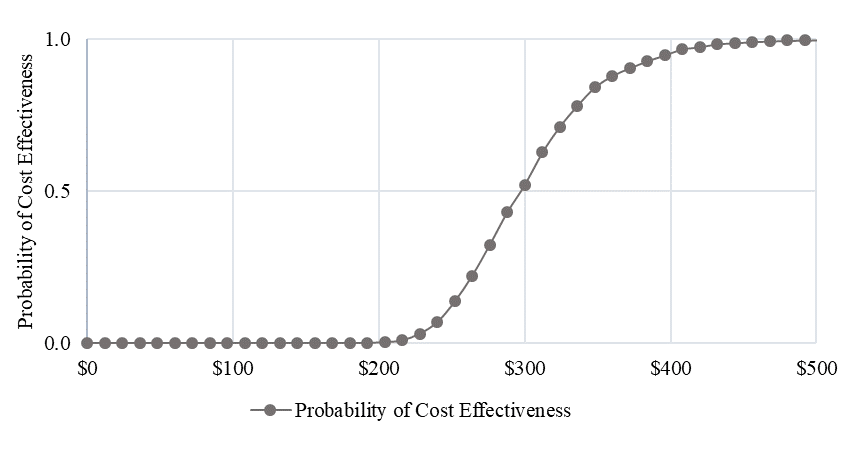

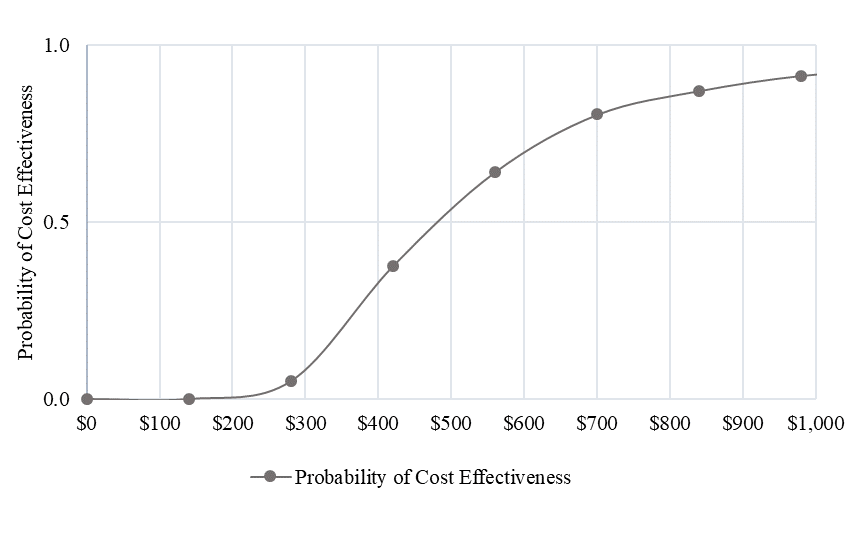

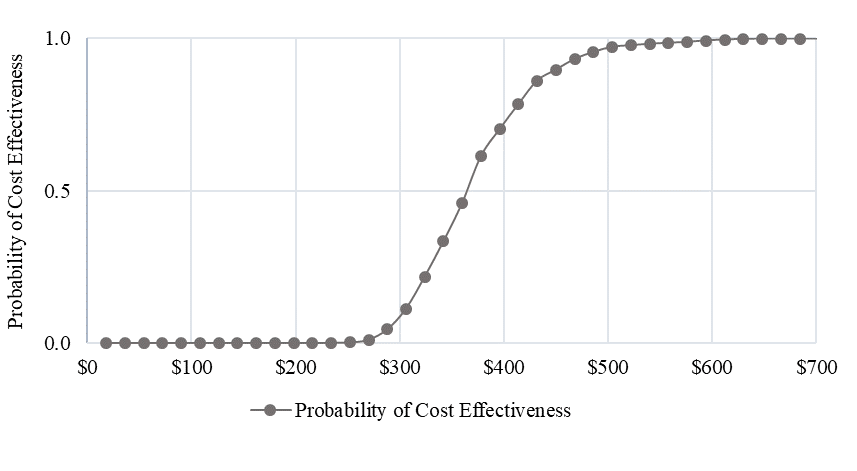


(c) Total referral acceptances

(b) Quitline referral acceptances

(a) Get Healthy service referral acceptances
